# From early risk to preventive action: Empowerment based digital triage and intervention for burnout, anxiety, and depression in Scandinavian healthcare workers

**DOI:** 10.3934/publichealth.2026029

**Published:** 2026-05-07

**Authors:** Clive Michelsen

**Affiliations:** Head of Research, Sciens College, Rödklintsgatan 2B, Tygelsjö 21873, Sweden

**Keywords:** burnout, anxiety, depression, Empowerment for Participation, early identification, healthcare workers, sick leave, Scandinavia, digital triage, artificial intelligence, occupational health, hospital management

## Abstract

**Background:**

Burnout, anxiety, and depression among healthcare workers are associated with long-term sickness absence, reduced quality of care, and impaired patient safety. In Sweden and other Scandinavian countries, common mental disorders account for a substantial share of sickness absence, with costs borne by employers and national insurance systems (the “payee side”). Early identification of workers at elevated risk, followed by proportionate preventive support, is therefore central to sustainable hospital management and occupational health. The Empowerment for Participation (EFP) assessment is a 110-item web-based system that maps everyday participation, demands, and self-expectations and derives validated risk indices for burnout, anxiety, and depression, together with constructs such as motivation, stress, and defence routines. The EFP battery has been used as a triage tool and an outcome framework in web-based psychotherapy trials targeting burnout risk and as a component in AI-driven digital triage concepts within the My-E-Health ecosystem. Prior Swedish evidence also links burnout and stress symptoms in healthcare workers to subsequent long-term sickness absence, supporting the relevance of risk indices for occupational prevention. Empowerment-related constructs have further been shown to be responsive to interventions such as mindfulness-based treatment.

**Objective:**

To integrate empirical data from a cohort of healthcare personnel assessed with the EFP battery into a broader preventive model linking early risk identification, digital and therapist-delivered support, and the Scandinavian sick-pay context, while clarifying the methodological limits of non-randomised, real-world data.

**Methods:**

I analysed EFP data from 325 healthcare workers with baseline (T1) and follow-up (T2) assessments who, in routine practice, either (1) received no structured treatment or AI support (no structured support/control), (2) engaged in human-delivered psychotherapy (human therapy), or (3) used an AI-supported, web-based intervention grounded in the EFP architecture (AI-only support). Outcomes included the EFP Empowerment index and EFP-derived risk indices for burnout, anxiety, and depression, plus stress burden, motivation, and defence routines. Change over time was examined within groups (paired t-tests; Cohen's d_z) and explored between groups using change-score ANOVAs (η²). Because groups were not randomised and differed at baseline, between-group comparisons were interpreted cautiously as descriptive.

**Results:**

At baseline, participants who went on to use human therapy or AI-only support showed higher EFP-derived risk for burnout, anxiety, and depression and lower empowerment than those who did not engage in structured support, consistent with risk-driven help-seeking. Under naturalistic conditions, human therapy and AI-only support were associated with large within-group improvements in empowerment and substantial reductions in EFP-derived risk indices, whereas the no-structured-support group showed only small changes. Defence routines decreased far more in the human therapy group than in the AI-only group, suggesting potentially distinct mechanisms. Improvements in empowerment were strongly associated with reductions in risk indices; however, the observational design did not support causal inference.

**Conclusions:**

The EFP battery functions as a practical framework for early risk identification and change-sensitive outcome monitoring that can support scalable digital triage and stepped-care pathways. However, because allocation to support was non-randomised and sickness absence was not measured in this cohort, claims about prevention of sick leave should be treated as hypotheses to be tested in prospective studies that include baseline-balanced comparisons and registry-based sick-leave endpoints.

## 1. Introduction

Health systems globally are grappling with rising levels of burnout, anxiety, and depression among healthcare professionals. In Scandinavian healthcare workers contribute to long-term sickness absence, poorer care quality, and patient-safety risks with costs to employers and insurers, making early identification and proportionate prevention through the 110-item EFP system—which generates validated risk and empowerment-related indices and has been used in web-based psychotherapy, AI triage, and intervention monitoring—highly relevant to sustainable occupational health and hospital management [1]–[7]. These conditions are increasingly understood not as individual “resilience failures” but as predictable outputs of chronically misaligned working conditions, high emotional load, and unresolved conflicts between external demands and internal standards [8]. Burnout is also consistently associated with impaired patient safety and lower quality of care, making it a workforce and governance issue as well as an individual health concern [9].

The associated sickness-absence burden is particularly visible in Scandinavian countries, where common mental disorders (CMDs; depression, anxiety, and stress-related disorders) account for a large and rising share of sickness absence diagnoses and disability pensions [1]. Long-term trajectories show that employees with sickness absence due to CMDs face increased risk of future recurrent absence and weakened labour market attachment [10]. These patterns have direct implications for the “payee side”, meaning the employers and social insurance systems that jointly finance sick pay and sickness benefits.

Under Swedish sick-pay rules, the first day of illness is an uncompensated waiting day, and from day 2 to day 14, the employer is responsible for sick pay. From day 15 onwards, the national Social Insurance Agency (Försäkringskassan) provides sickness benefit, typically at a lower replacement rate [11]. This structure creates shared economic incentives for early detection, rapid support, and prevention of progression from early psychological strain to long-term work incapacity.

Within hospitals and regional healthcare systems, managers therefore face a multi-layered challenge: how to detect workers drifting into high-risk states before sickness absence escalates; how to triage them to appropriate support; and how to do so at scale without overwhelming traditional occupational health resources. Evidence linking burnout to subsequent long-term sickness absence among Swedish healthcare workers is robust [6]. Additionally, work similarly shows that early stress and depressive symptoms among healthcare workers are associated with prolonged sickness absence [12].

Against this background, generic screening tools and stand-alone symptom questionnaires are insufficient. Organisations need instruments that can (1) identify risk before breakdown, (2) explain risk in contextual and actionable terms, and (3) provide reliable outcome metrics for interventions that may be digital, therapist-delivered, or blended.

The Empowerment for Participation (EFP) assessment was developed to meet this need. EFP is a 110-item web-based, visual-analogue instrument that maps motivation, stress, defence routines, engagement, and multiple context domains (e.g., workload, role clarity, self-expectations, social environment, and health) and uses these data to derive validated risk indices for burnout, anxiety, and depression [2],[3].

The EFP battery has also served as the backbone for web-based psychotherapy trials targeting burnout risk [4] and for digital triage concepts in the My-E-Health ecosystem [5]. Related work further supports the responsiveness of empowerment-related constructs to interventions such as mindfulness-based treatment [7].

In this paper, I integrate a new cohort of EFP data from healthcare workers into this wider framework. I (1) re-analysed EFP data from 325 healthcare personnel who completed the full 110-item assessment at baseline and follow-up and who received either no structured support, human therapy, or AI-only digital support; (2) interpreted the findings through the lens of early identification and stepped prevention; and (3) situated the findings within Scandinavian sick-pay incentives and hospital management concerns such as retention, quality of care, and patient safety. Throughout, I explicitly acknowledge that the dataset reflects naturalistic, non-randomised care pathways and therefore supports cautious interpretation.

## 2. Theoretical and empirical background

### 2.1. Burnout, anxiety, depression, and sick leave in Scandinavian healthcare

In Nordic welfare states, sickness absence is a health indicator and a large, visible cost line. Mental disorders are the leading diagnostic group underlying long-term sickness absence and disability pensions, particularly among younger workers and women [1]. For healthcare workers, high workload, emotional labour, and shift work further amplify vulnerability to burnout and CMD-related sickness absence [6].

Peterson's work on stress and burnout in Swedish healthcare workers showed that burnout co-occurs with poor self-rated health, elevated depression, and anxiety, and prospectively predicts long-term sickness absence [6]. In a study of more than 3700 employees in a Swedish county council, constellations of work characteristics (e.g., high demands, low influence, and lack of support) differentiated burnout profiles and were associated with different patterns of sickness absence and self-rated health [13]. More recent Scandinavian studies reaffirm that early stress and depressive symptoms among care workers predict prolonged sickness absence and impaired working-life trajectories [12].

Parallel evidence from clinical and quality-of-care research shows that burnout in physicians and nurses is linked to increased patient-safety incidents and lower quality of care. Moreover, meta-analyses of physician burnout demonstrate associations with increased risk of safety incidents, poorer professionalism, and lower patient satisfaction [14]. A meta-analysis of nurse burnout reports consistent links with poorer care quality and lower patient satisfaction [15]. Together, this evidence indicates that burnout, anxiety, and depression constitute a nexus of risk spanning individual well-being, sick-leave costs, and patient-facing harms.

### 2.2. The EFP assessment as a risk engine

The EFP framework was developed to capture this nexus in a single integrated instrument. Two features are central. First, the EFP battery provides comprehensive contextualisation: 110 items cover work demands, role clarity, social and family context, physical health, self-esteem, participation, and perceived influence [2],[3]. Second, the EFP includes derived risk indices for burnout, anxiety, and depression and complementary constructs (motivation, stress burden, defence routines, engagement) that can indicate why a given risk profile may be maintained and where intervention may be most actionable [2].

In “Measuring Employee Risk for Burnout”, Michelsen reported that the EFP-derived burnout risk index predicts sickness presence, work-related stress, and engagement and that validated risk categories (minimal, low, moderate, high, very high) discriminate meaningful differences in employee well-being and performance [3]. The companion paper further validates the EFP battery and reports acceptable to excellent reliability for its scales, including patterns in which high stress and defence routines combined with low empowerment flag risk for burnout, anxiety, and depression [2].

In a controlled trial of web-based psychotherapy to treat and prevent burnout, the EFP battery was a triage tool and an outcome framework. Participants with elevated burnout risk engaged in a CBT-informed online protocol, and results showed substantial reductions in EFP-derived burnout risk and high reliability of the EFP risk indices over time [4]. Related work on mindfulness-based treatment in psychiatric care supports the broader proposition that empowerment-related constructs are measurable and responsive to intervention [7].

### 2.3. Digital triage, AI, and My-E-Health

The digital health platform My-E-Health has been used as an implementation environment for the EFP battery, combining web-based assessments, asynchronous and synchronous therapy, and digital coaching. In a 2025 perspective article, Michelsen and Kjellgren outline an AI-driven digital triage model in which assessment data, including EFP-type inputs, are used to route users to an appropriate level of care and to monitor treatment response over time [5].

In this model, triage is built around (a) a continuous, low-friction assessment; (b) automated risk stratification for burnout, anxiety, and depression; (c) rule-based and AI-assisted routing to self-help tools, web-based therapy, or higher-intensity human care; and (d) use of empowerment and symptom measures as feedback signals for adaptive treatment [5]. Such triage architectures may be particularly relevant for hospital settings where occupational health resources are constrained, and workforces are distributed across units and shifts.

The model also fits the preventive orientation of Scandinavian social insurance systems, which increasingly emphasise early action to prevent long-term work incapacity [10]. In this paper, I illustrate how these ideas may operate in practice using a new cohort of healthcare worker EFP data.

## 3. Methods

### 3.1. Design and setting

This study is a naturalistic, non-randomised analysis of routine practice data collected in the My-E-Health ecosystem. The analysis focused on pre-post change in EFP-derived indices between baseline (T1) and follow-up (T2). Because participants self-selected (or were organisationally routed) into different support pathways, group comparisons reflected real-world patterns rather than experimentally controlled effects.

### 3.2. Participants and grouping

The dataset consisted of 325 healthcare workers who completed the full 110-item EFP assessment at T1 and T2. Participants represented a range of healthcare roles (e.g., physicians, nurses, allied health professionals, and administrators) in hospital or hospital-adjacent settings. The mean age was in the mid-forties with an approximately even gender distribution.

Participants were classified into support groups based on documented engagement between T1 and T2. The no structured support group (control; n = 253) completed EFP assessments at T1 and T2 but did not engage in documented psychotherapy or AI-only digital support in the interim. The human therapy group (n = 38) received structured psychotherapy (often CBT-informed, sometimes blended with other modalities) via the platform and/or affiliated providers, with scheduled human contact. The AI-only support group (n = 34) engaged with AI-supported, web-based tools derived from the My-E-Health architecture without registered human psychotherapy during the interval.

A fourth category (“human therapy + AI”) was coded in the platform but had no analysable cases with complete EFP data in this cohort. Baseline EFP profiles differed across groups; those who engaged in human therapy or AI-only support tended to show higher baseline risk and lower empowerment than the control group, which is consistent with risk-driven help-seeking and is central to the interpretive limits of the dataset.

### 3.3. Intervention descriptions

Human therapy consisted of clinician-delivered psychotherapy sessions, typically CBT-informed, delivered through the My-E-Health ecosystem and/or affiliated providers. The EFP assessment was used for assessment-driven feedback and progress monitoring.

AI-only support referred to platform-delivered, fully digital support modules. During the study period, these modules included structured CBT-informed exercises, psychoeducation, reflective journaling prompts, and automated feedback based on EFP scoring outputs (risk indices, empowerment, and related constructs). The automated feedback was generated by algorithmic decision rules anchored in the EFP scoring architecture (i.e., rule-based personalisation of feedback and module recommendations) rather than by an autonomous generative large language model. No human psychotherapy sessions were registered for participants in this group during the interval. The analytic dataset did not include detailed usage logs; therefore, intervention “dose” (time on platform, module completion) could not be modelled.

### 3.4. Measures

From the 110 EFP items and their established scoring algorithms, I extracted the following outcomes: empowerment index, burnout risk index (external demands-self-expectations conflict; ED-SE), anxiety risk index, depression risk index, stress burden, defence routines, and motivation.

The burnout risk index was computed from 30 items focusing on external demands, self-expectations, and their conflict and was validated as an indicator of burnout risk with interpretable risk bands (minimal, low, moderate, high, very high) [2]–[4]. Anxiety and depression risk indices were similarly derived from specific EFP item constellations rather than generic symptom checklists, supporting early identification by embedding risk in the person's participation context [2]–[4].

### 3.5. Statistical analysis

Change scores are reported as Δ = T2–T1. Within each group, I tested pre-post change using paired t-tests and reported Cohen's d_z (paired-samples effect size). Between groups, I explored differences in change scores using one-way ANOVA, and reported η² as a measure of variance explained. Because groups were not randomised and differed at baseline, between-group ANOVA results were interpreted as descriptive and potentially confounded by selection effects and regression to the mean. Finally, I examined correlations between Δ empowerment and changes in risk indices and related constructs to evaluate whether empowerment behaves as a hub variable; these correlations were interpreted as associations rather than evidence of causality.

## 4. Results

### 4.1. Baseline risk profiles and selection into support

At baseline, EFP-derived risk indices and related constructs were higher among participants who subsequently engaged in human therapy or AI-only support compared with those who did not engage in structured support. This pattern is consistent with risk-driven help-seeking and supports the interpretation of the EFP battery as an early identification framework in routine practice [3]. This also means that the no-structured-support group should be understood as a lower-risk comparison group rather than as an untreated high-risk control group.

### 4.2. Pre-post change in empowerment and risk indices

Table 1 summarises pre-post change in empowerment and the EFP-derived risk indices. In the no-structured-support group, changes were small: empowerment increased modestly (Δ = +13.5; d_z = 0.20; p = 0.001) and burnout risk decreased modestly (Δ = −10.0; d_z = −0.20; p = 0.002), while anxiety risk did not change significantly (p = 0.13) and depression risk showed a small reduction (Δ = −3.7; d_z = −0.14; p = 0.030).

In contrast, human therapy was associated with large within-group improvements across all indices: empowerment increased (Δ = +93.6; d_z = 0.90; p < 0.001) and burnout, anxiety, and depression risk indices decreased substantially (Δ = −70.6; d_z = −1.14; Δ = −44.7; d_z = −0.98; and Δ = −37.8; d_z = −0.89, respectively; all p < 0.001).

AI-only support showed a similar pattern of large within-group improvement: empowerment increased (Δ = +83.4; d_z = 0.70; p < 0.001) and burnout, anxiety, and depression risk indices decreased (Δ = −59.1; d_z = −0.80; Δ = −31.5; d_z = −0.71; and Δ = −30.1; d_z = −0.73, respectively; all p < 0.001).

Exploratory ANOVAs on change scores indicated that support type explained a non-trivial proportion of variance in change for empowerment and each risk index (η² approximately 0.14 to 0.16). Because baseline risk and empowerment differed across groups, these between-group effects should be interpreted as descriptive and potentially inflated by regression to the mean; they do not establish causal efficacy.

**Table 1.** EFP empowerment index and EFP-derived risk indices: pre-post change by support pathway.

Outcome	Group	n	Pre M (SD)	Post M (SD)	Δ (Post-Pre) M (SD)	Cohen's d_z	t (df)	p
Empowerment index	No structured support	253	751.3 (120.06)	764.8 (122.92)	+13.5 (66.29)	0.20	3.25 (252)	p = 0.001
	Human therapy	38	698.7 (116.32)	792.3 (113.34)	+93.6 (103.97)	0.90	5.55 (37)	p < 0.001
	AI-only support	34	718.9 (165.55)	802.2 (167.28)	+83.4 (118.85)	0.70	4.09 (33)	p < 0.001
Burnout risk (ED-SE conflict)	No structured support	253	166.09 (89.92)	156.10 (90.50)	−10.0 (50.35)	−0.20	−3.16 (252)	p = 0.002
	Human therapy	38	201.34 (79.16)	130.76 (76.96)	−70.6 (61.66)	−1.14	−7.06 (37)	p < 0.001
	AI-only support	34	190.35 (108.29)	131.26 (104.58)	−59.1 (73.74)	−0.80	−4.67 (33)	p < 0.001
Anxiety risk	No structured support	253	104.3 (57.4)	101.3 (57.7)	−3.0 (57.7)	−0.10	−1.53 (252)	p = 0.13
	Human therapy	38	131.8 (54.6)	87.1 (49.6)	−44.7 (49.6)	−0.98	−6.05 (37)	p < 0.001
	AI-only support	34	125.1 (69.3)	93.6 (70.8)	−31.5 (70.80)	−0.71	−4.14 (33)	p < 0.001
Depression risk	No structured support	253	98.6 (49.9)	94.9 (51.7)	−3.7 (51.7)	−0.14	−2.18 (252)	p = 0.030
	Human therapy	38	121.3 (45.9)	83.5 (45.7)	−37.8 (45.7)	−0.89	−5.47 (37)	p < 0.001
	AI-only support	34	116.3 (68.6)	86.2 (67.1)	−30.1 (67.1)	−0.73	−4.25 (33)	p < 0.001

Note: Δ = T2–T1. Cohen's d_z is the paired-samples (within-group) effect size. ED-SE = external demands-self-expectations.

### 4.3. Stress burden, defence routines, and motivation

Within-group effect sizes for stress burden, defence routines, and motivation are shown in Table 2. The largest divergence between human therapy and AI-only support was observed for defence routines (d_z = −1.73 in human therapy vs d_z = −0.60 in AI-only support, compared with d_z = −0.04 in the no-structured-support group). This suggests that while AI-only support may be sufficient for reducing symptom-linked risk indices for many participants, human-delivered therapy may provide additional leverage for addressing entrenched defensive patterns.

**Table 2.** Within-group pre-post effect sizes (Cohen's d_z) for empowerment, risk indices, and related constructs.

Outcome	No structured support	Human therapy	AI-only support
Empowerment	+0.20	+0.90	+0.70
Burnout risk (ED-SE conflict)	−0.20	−1.14	−0.80
Anxiety risk	−0.10	−0.98	−0.71
Depression risk	−0.14	−0.89	−0.73
Stress burden	−0.14	−0.79	−0.61
Defence routines	−0.04	−1.73	−0.60
Motivation	+0.10	+0.86	+0.56

Note: Negative d_z values indicate reductions from T1 to T2; positive values indicate increases.

### 4.4. Associations between empowerment change and risk reduction

Across the sample, greater gains in empowerment were associated with larger reductions in burnout, anxiety, and depression risk indices and with improvements in stress burden, defence routines, and motivation. These associations are consistent with empowerment functioning as a hub construct in the EFP framework. However, because all measures were collected in the same observational design and time window, the correlations cannot be interpreted as demonstrating that empowerment causes risk reduction; reciprocal relationships and shared change processes remain plausible explanations [7].

## 5. Discussion

### 5.1. What the findings do and do not suggest

This cohort shows two patterns that are jointly important for occupational mental health practice. First, higher baseline EFP risk and lower empowerment were concentrated among those who subsequently engaged with human therapy or AI-only support, which is consistent with the EFP battery functioning as an early identification framework in real-world help-seeking contexts [3]. Second, large within-group improvements were observed in both supported groups, while changes in the no-structured-support group were small. These patterns are compatible with a preventive, empowerment-oriented model, but the design does not permit strong causal conclusions about intervention effectiveness because allocation was non-randomised, and baseline profiles differed substantially.

The baseline non-equivalence is not a minor methodological detail; it is central to interpretation. The no-structured-support group was not an untreated high-risk group but a lower-risk group that did not engage in structured support. Therefore, between-group comparisons likely overstate the apparent impact of support pathways, in part due to selection effects and regression to the mean. Thus, I reframed between-group analyses as exploratory and emphasised within-group change patterns, while recommending prospective designs with baseline balancing (randomisation, matching, or quasi-experimental designs) in future work.

### 5.2. Implications for the Scandinavian sick-leave context

The Scandinavian sick-pay context provides a strong rationale for early action. In Sweden, employers cover sick pay for the first two weeks, after which national insurance assumes responsibility [11]. This division of costs creates shared incentives on the “payee side” (employers and insurers) to prevent escalation from early psychological strain to longer-term work incapacity. However, sickness absence days were not measured in this cohort. Therefore, the data do not demonstrate reductions in sick leave; rather, they show that EFP-derived risk indices and empowerment, which have theoretical and empirical links to work functioning and sickness absence risk, shift in favourable directions when support is engaged [6].

A key next step is to test whether EFP risk trajectories predict and mediate changes in objectively measured sickness absence (registry or employer-recorded days) and return-to-work outcomes in Scandinavian settings. Such work would enable the proposed prevention model to be evaluated directly against the outcomes that matter most for workforce sustainability and the payee side.

### 5.3. Hospital healthcare management and patient safety

For hospital management, the stakes are not just absenteeism but also quality and safety. Evidence linking burnout to patient safety incidents is overwhelming: meta-analyses show that burnout in physicians and nurses is associated with higher rates of safety incidents, poorer care quality, and lower patient satisfaction [14].

An EFP-driven prevention framework can be incorporated into hospital governance as follows:

• *Risk dashboards:* Aggregated EFP data at the unit or hospital level can form risk heatmaps, highlighting departments where burnout and CMD risk are rising alongside high demands and low empowerment.

• *Targeted interventions:* High-risk units can receive tailored interventions (e.g., increased supervision, workload adjustments, and team coaching) in addition to individual-level therapy and AI-support.

• *Patient safety integration:* Risk indices can be cross-tabulated with internal patient safety indicators, enabling managers to track whether reductions in staff burnout risk coincide with reductions in safety incidents.

Such integration is particularly important given contemporary concerns about staff fatigue and patient safety in health systems, including recent warnings about fatigue-related errors and adverse events in national healthcare services [16]. Although these examples are drawn from the UK rather than Scandinavia, the underlying dynamics, overwork, chronic fatigue, and risk to patient safety, are similar.

### 5.4. AI-supported interventions versus human therapy: distinct mechanisms

One of the most clinically and organisationally relevant findings is the differential change in defence routines. Human therapy was associated with a very large reduction in defence routines (d_z = −1.73), whereas AI-only support showed a moderate reduction (d_z = −0.60), and the no-structured-support group showed minimal change. Defence routines in the EFP framework represent rigid protective patterns (e.g., denial, avoidance, and externalisation) that may maintain risk over time by limiting emotional processing and behavioural flexibility [2].

This divergence suggests a plausible division of labour in stepped care. AI-only support may be particularly effective for structured skills practice, psychoeducation, and self-reflective routines (e.g., CBT exercises, journaling, and behavioural activation), which can reduce symptom-linked risk indices for many users. Human therapy may offer additional leverage for addressing entrenched defensive patterns through relational processes such as alliance, interpersonal feedback, and contextual meaning-making. These hypotheses should be tested directly (e.g., by modelling defence routines as moderators and mediators of treatment response), but they provide a practical foundation for blended pathways in which EFP profiles inform triage to AI-only, blended, or therapist-led care [5].

### 5.5. Implementation considerations

If EFP-based screening and triage are implemented in healthcare organisations, governance and trust are critical. Staff must trust that EFP data are used to support, not penalise, individuals. Clear data governance should separate individual-level clinical data from managerial use, with consent processes and privacy safeguards aligned with applicable data protection requirements.

At the organisational level, aggregated EFP indicators could support unit-level risk monitoring (e.g., identifying departments where burnout risk and low empowerment are rising) and guide targeted organisational interventions (workload adjustments, team-based support, leadership development). Any such use should be carefully designed to avoid re-identification and to ensure that aggregated monitoring is coupled with tangible preventive action rather than surveillance.

### 5.6. Limitations of my dataset

Several limitations constrain interpretation. First, allocation to support pathways was non-randomised, and groups differed substantially at baseline, making causal inference inappropriate and raising the likelihood of regression-to-the-mean effects. In addition, within-group effect sizes in naturalistic pre-post designs can be inflated relative to randomised trials because they also capture non-specific change processes (e.g., spontaneous recovery, measurement reactivity, and expectancy effects). Second, the cohort does not include objective sickness absence outcomes; the paper's sick-pay discussion is therefore conceptual, grounded in the Scandinavian policy context and evidence linking burnout and CMD symptoms to sickness absence [6],[12]. Third, intervention dose and engagement metrics were not available, limiting the ability to estimate dose-response relationships or to distinguish high- from low-engagement users within the AI-only pathway. Finally, the cohort reflects one digital ecosystem (My-E-Health) and may not generalise to settings with different occupational health infrastructures or different AI implementations.

### 5.7. Future research directions

In future studies, researchers should link EFP trajectories to registry-based sickness absence and return-to-work outcomes in Scandinavian contexts, enabling direct tests of predictive validity, mediation, and economic impact. They should also evaluate baseline-balanced comparisons (randomised or quasi-experimental designs) and explicitly model moderators such as baseline defence routines and motivation to optimise triage decisions [5]. Finally, unit-level intervention studies are needed to determine whether aggregated EFP risk signals can guide organisational changes that improve staff outcomes and patient safety over time.

## 6. Conclusion

Burnout, anxiety, and depression among healthcare workers represent converging risks for staff well-being, healthcare quality, patient safety, and the sustainability of Scandinavian sick-pay systems. In this naturalistic cohort, higher baseline EFP risk concentrated among those who engaged in support, and human therapy and AI-only support were associated with substantial improvements in empowerment and reductions in EFP-derived risk indices, while the no-structured-support group showed only small changes. These findings support the EFP battery as a practical framework for early identification and outcome monitoring that can inform stepped, scalable support pathways. Furthermore, the non-randomised design and the absence of sickness absence endpoints require cautious interpretation; rigorous prospective studies are needed to test whether EFP-guided support reduces objective sick leave and improves organisational outcomes.

## Use of AI tools declaration

The authors declare they have not used Artificial Intelligence (AI) tools in the creation of this article.
